# Key concepts for improving primary care diagnosis in Japan: Diagnostic error in primary health care

**DOI:** 10.1002/jgf2.228

**Published:** 2018-12-18

**Authors:** Takashi Watari

**Affiliations:** ^1^ Postgraduate Clinical Training Center Shimane University Hospital Shimane Japan

To the Editor:

Diagnostic error is one of the most important considerations for primary care physicians. Recently, a study indicated many currently harmful aspects of primary care settings: the mortality rate, medical economical problem, medical malpractice claims, etc.^1^, ^2^ In Japan, most primary care physicians are mainly responsible for diagnosis with inadequate clinical settings such as clinics. Therefore, primary care physicians face multiple difficulties regarding diagnostic errors: Primary care physicians are often the first‐contact physician; usually work alone in many clinical settings in Japan; and not only diagnose and treat the patient, but also manage comprehensive and coordinated care.

As mentioned above, diagnostic error is a very important concern for us. However, physicians’ interest in the primary care field is insufficient and there has been difficulty conducting clinical research on diagnostic error in our nation. I believe there are several reasons for these challenges, one being the extreme difficulty in defining diagnostic error. According to the previous guidelines, a diagnostic error can be traditionally considered as “misses,” wrong,” or “delays”^3^. In addition, it is possible that “over diagnosis” will be included as a new definition from the Choosing Wisely campaign, which is the diagnosis of a condition that, if unrecognized, would not cause symptoms or harm a patient.^4^ Another reason is the unique cultural background of Japan. Diagnostic error is a very common clinical outcome in primary care fields; the previous guidelines reported that “to error is human” and no one inevitable to avoid this risk with cognition biases. However, due to our deep and strong mindset in Japanese culture, making mistakes is associated with shame; we tend to avoid discussion about the process of errors in diagnosis and hide the precious experience in depths of our hearts. Without conscious deliberation, this mindset may lead us to miss important opportunities to learn from our own mistakes.

Based on these points, I suggest the following: (a) including scope for reflective education on diagnostic errors in medical school; (b) promoting clinical research on diagnostic errors in Japan; (c) establishing a nationwide data bank of diagnostic errors; (d) collaborating with the field of patient safety, the Choosing Wisely campaign, and activities of the Ministry of Health, Labour and Welfare in Japan; and (e) promoting cooperation of doctors, paramedical staff, and patients. Consequently, I believe that as top priorities, we need to launch a new program for clinical practice and the education system in this new field focusing on diagnostic error for primary care physicians, as well as to enhance clinical research on diagnostic errors that can harm patients in clinical practice.

## FUNDING INFORMATION

Author T.W. was supported by grants by JSPS KAKENHI Grant Number 17K15745.

## CONFLICT OF INTEREST

The authors have stated explicitly that there are no conflicts of interest in connection with this article.
